# Optimal Follow-Up Duration for Assessment of Birth Defects After *In Vitro* Fertilization–Embryo Transfer: A Multicenter 5-Year Cohort Study in China

**DOI:** 10.3389/fendo.2022.817397

**Published:** 2022-03-18

**Authors:** Chun-Lin Liu, Ping Li, Gui-Feng Cai, Abraham Morse, Jun Liu, Zhi-Heng Chen, Xiu Zhang, Ling Sun

**Affiliations:** ^1^Center for Reproductive Medicine, Guangzhou Women and Children’s Medical Center, Guangzhou Medical University, Guangzhou, China; ^2^Reproductive Medicine Center, Jiangmen Central Hospital, Affiliated Hospital of Sun Yat-sen University, Jiangmen, China; ^3^Reproductive Medicine Center, Zhuhai Maternal and Child Healthcare Hospital, Zhuhai, China; ^4^Department of Obstetrics and Gynecology, Guangzhou Women and Children’s Medical Center, Guangzhou, China; ^5^Department of Obstetrics and Gynecology, Tufts Medical School, Boston, MA, United States

**Keywords:** birth defects, cohort study, *in vitro* fertilization (IVF), follow-up duration, loss to follow-up (LTFU)

## Abstract

**Objective:**

To investigate the impact of a 5-year follow-up on the incidence of identified birth defects in children conceived using assisted reproductive technologies (ART).

**Methods:**

A 5-year cohort study was performed in three ART centers from January 2013 to October 2018. 1,543 women with 1,985 infants who delivered successfully or underwent termination of pregnancy due to malformations were recruited in this study. Follow-up was conducted by phone interview, 7 days, 1 year, 3 years, and 5 years after birth. Collected data included whether one or more birth defects were diagnosed, the category of birth defects, and when the malformation was diagnosed. Cumulative incidence of birth defects and the loss to follow-up rate of each follow-up was compared.

**Results:**

According to the diagnostic criterion of birth defects, 111 cases of one or more birth defects were recorded, with a total of 117 birth defects after the 5-year follow-up. 0.2% (4/1,985) of birth defects were diagnosed before delivery; 2.7% (54/1,985) at 7 days; 5.0% (100/1,985) after 1 year; 5.5% (109/1,985) after 3 years; and 5.6% (111/1,985) after 5 years. 3.4% (4/117) of defects were diagnosed prenatally, 45.3% (53/117) of defects diagnosed within the first 7 days after delivery, 40.2% (47/117) diagnosed during 7 days to 1 year, and 9.4% (11/117) of defects diagnosed in 1–3 years after birth. The remaining 1.7% (2/117) of defects were diagnosed between the ages of 3 and 5 years. Among the 1,543 patients, 99.9% patients (1,542/1,543) responded to the telephone interview at 7 days after delivery; the response rate was 89.0% (1,373/1,543) at 1 year, 81% (1,250/1,543) at 3 years, and 64.5% (995/1,543) after 5 years.

**Conclusion:**

We suggest that in ART, 1-year follow-up should be the minimum requirement and 3-year follow up the optimal length of follow-up that balances resource requirements with ascertainment completeness.

## Introduction

Since the first *in vitro* fertilization (IVF) baby was born in 1978, there has been a rapid increase in the number of couples using assisted reproductive technology (ART). It was estimated that ART has resulted in hundreds of thousands of infants born globally every year (1). With the increasing number of children conceived using ART, their long-term health, particularly the incidence and types of birth defects, had been an important concern for society.

Birth defects are defined as structural or functional anomalies (for example, metabolic disorders) that occur during intrauterine life and are identified prenatally, at birth, or sometimes are only detected later in infancy, such as hearing defects (2).

The prevalence of birth defects after ART varies from 1% to 6.2% according to different studies (3–8). The inconsistency in the overall rate of birth defects reported may be due to the differences in surveillance methods and birth defect definitions used by the authors, particularly inconsistent exclusion and inclusion criteria regarding what constitute minor and major defects or variants. In addition, the duration of follow-up may be another important factor which affects reported birth defect rates. Compared to studies with at least 1 year of postnatal follow-up (8, 9), studies (3, 5, 6) with a short follow-up (e.g., only up to 6 weeks after delivery) appeared to report lower birth defect rates which may be due to undercounting of defects (e.g., nervous system malformations) that are difficult to ascertain shortly after birth.

In theory, the incidence of birth defects increases with longer follow-up. However, there is no consensus regarding follow-up duration with regard to capturing all, or nearly all, defects. For example, birth defects such as septate uterus, the most common uterine anomaly, with an estimated prevalence of 2%–3% of women, cannot be diagnosed until reproductive age (10). Several studies investigated the necessity of prolonged follow-up duration for the surveillance of birth defects (11, 12) and found that the rate of birth defects increases gradually, from 2.2% at birth to 4% at 1 year, to 5.2% at 3 years and 6% at 5 years (13, 14). In order to maximize recognition of birth defects, including many urogenital defects, as well as central nervous system, musculoskeletal, and cardiovascular defects, it has been suggested that studies of birth defect rates should commit to up to 6 years of follow-up (11).

However, extending the follow-up duration means more resources would be required to maintain a registry or complete a study. In addition, as follow-up intervals lengthen, the loss to follow-up (LTFU) rate increases which may affect the accuracy of estimates. Therefore, it may be useful to identify where the balance lies between identifying more birth defects and the resources required to conduct research.

The aim of this study was to gain a better understanding of which defects are diagnosed and when defects are diagnosed after ART treatment, and to document the trajectory of LTFU rates. We conducted a multicenter cohort study with 5-year follow-up of all infants conceived using ART in Guangdong Province, China, to investigate the prevalence of all-cause and specific cause of birth defects.

## Materials and Methods

### Study Design and Patients

This was an observational, longitudinal, multicenter, study. Participants were recruited from January 2013 to December 2015 from three reproductive centers in Guangdong, China. The study was approved by the Reproductive Medical Ethics Committee of Guangzhou Women and Children’s Medical center.

### Data Collection

According to a birth defect report of China in 2012 (15), women who had undergone *in vitro* fertilization (IVF) and/or intracytoplasmic sperm injection (ICSI) and had reached 28 weeks of pregnancy were invited to participate in this study. These couples were informed and signed a follow-up consent. Telephone interviews were conducted four times: 7 days, 1 year, 3 years, and 5 years after delivery. At the first interview (7 days after delivery), the maternal complications, neonatal complications, category and quantity of birth defects, and loss to follow-up information were recorded; meanwhile, terminations for birth defects and stillbirths of over 28 weeks of gestation were recorded as well. At the following three times, only birth defects and loss to follow-up data were included (**Supplement 1**).

All researchers were trained to assure consistent language during follow-up calls using a standard questionnaire (**Supplement 1**). At each follow-up time, if the first call was not answered, a maximum of two additional calls was made at 3–4-day intervals. Loss to follow-up was defined as no response after three calls or a non-working phone number.

### Definition of Birth Defects

Defects were classified (coding: Q00-99) according to the International Classification of Diseases and Health Related Problems, 10th Revision (ICD Manual, 1992) (16). Birth defects include structural abnormalities, biochemical abnormalities, and those that are chromosomal or otherwise genetic abnormalities. Minor defects are generally excluded from the registry, with the exception of those that require treatment or are disfiguring. The incidence of birth defects was defined as the total number of births with at least one defect/total number of births after 28 weeks of pregnancy.

### Statistical Analysis

Categorical variables were expressed as frequency and percentage and were compared between groups by means of the Pearson chi-square test or Fisher exact test. A result was considered statistically significant if a two-tailed *p* value was less than 0.05. All data analyses were performed using SPSS for windows 20.0 (IBM, Armonk, NY).

## Results

### Assessment of the Loss to Follow-up Rate

**Figure 1:**
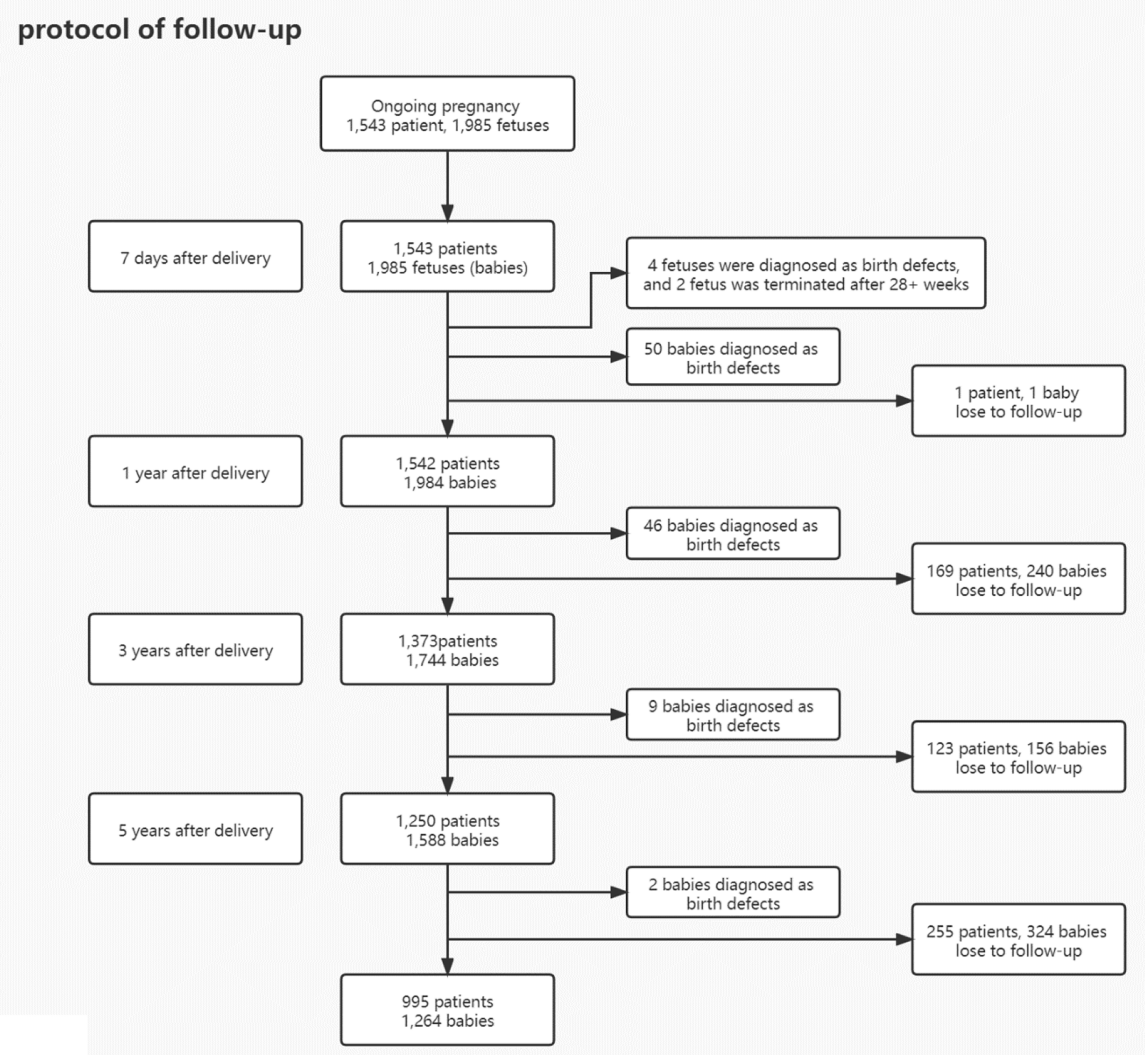
Protocol of follow-up.

A total of 1,543 women and 1,985 fetuses were enrolled in this study. 99.9% of patients (1,542/1,543) responded to a request for telephone interview at 7 days after delivery. The response rates were 89.0% (1,373/1,543) at 1 year; 81% (1,250/1,543) after 3 years; and 64.5% (995/1,543) after 5 years (**Figure 1**).

### Cumulative Prevalence of Birth Defects

**Figure 2:**
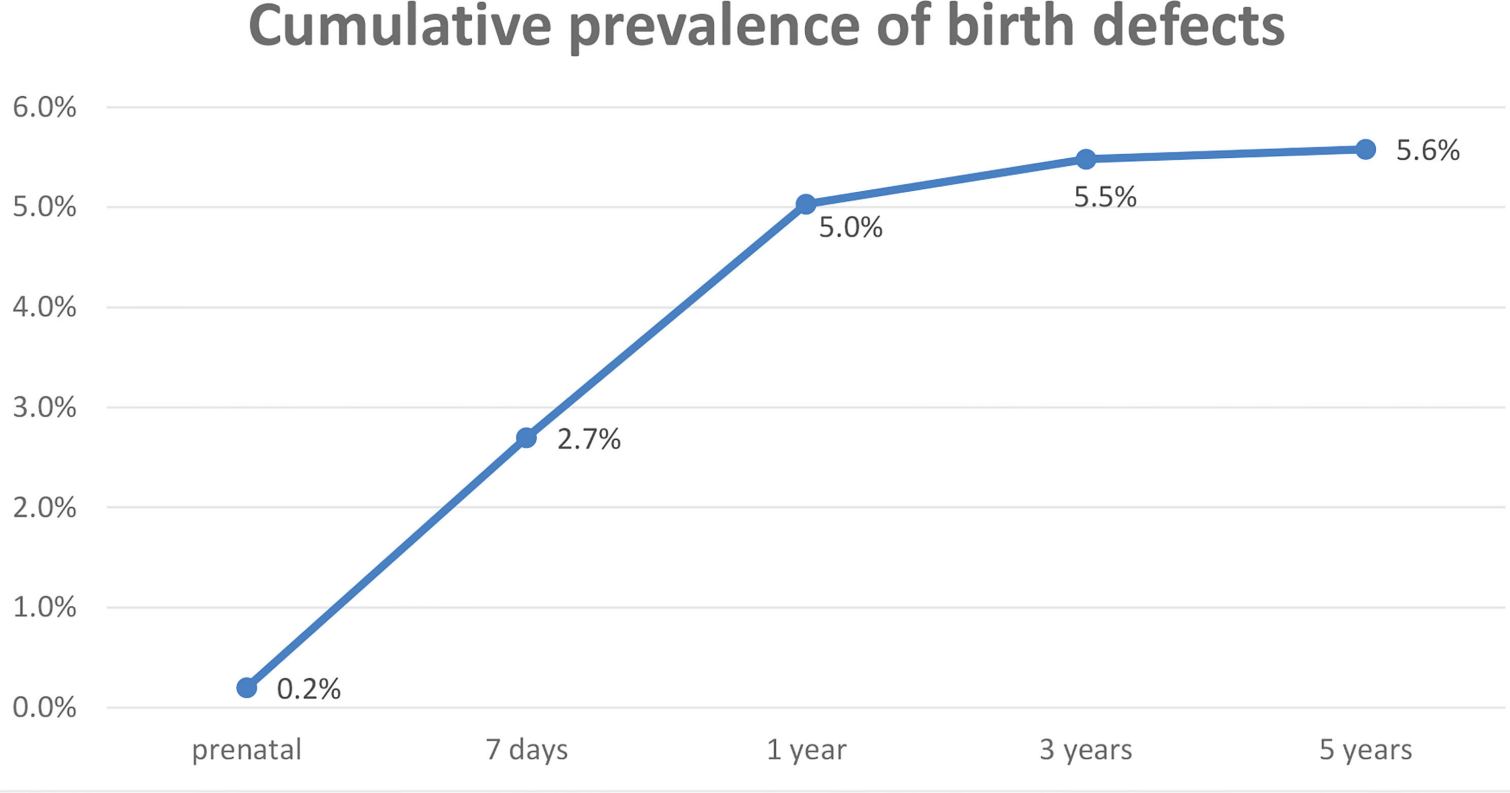
Cumulative prevalence of birth defects in different follow-up durations.

According to the diagnostic criterion of birth defects, 1,985 28-week-old fetuses were recruited into the study. Among them, 111 cases of one or more birth defects were recorded, with a total of 117 birth defects after the 5-year follow-up. The cumulative incidence of birth defects diagnosed was 0.2% (4/1,985) before delivery; 2.7% (54/1,985) 7 days after delivery; 5.0% (100/1,985) after 1 year; 5.5% (109/1,985) after 3 years; and 5.6% after (111/1,985) after 5 years (**Figure 2**).

### Classification of Birth Defects

3.4% (4/117) of defects were diagnosed prenatally, and 45.3% (53/117) of defects were diagnosed within the first 7 days after delivery. 40.2% (47/117) were first recorded at the 1-year follow-up, and 9.4% (11/117) of defects were first recorded at the 3-year follow-up. The remaining 1.7% (2/117) of defects were first recorded at the 5-year follow-up.

Of the 4 fetuses that were diagnosed with congenital malformations before delivery, two fetuses underwent termination of pregnancy (lack of right kidney or right subependymal hemorrhage) and the other two fetuses (tetralogy of Fallot and congenital hydronephrosis) received treatment after delivery.

**Figure 3:**
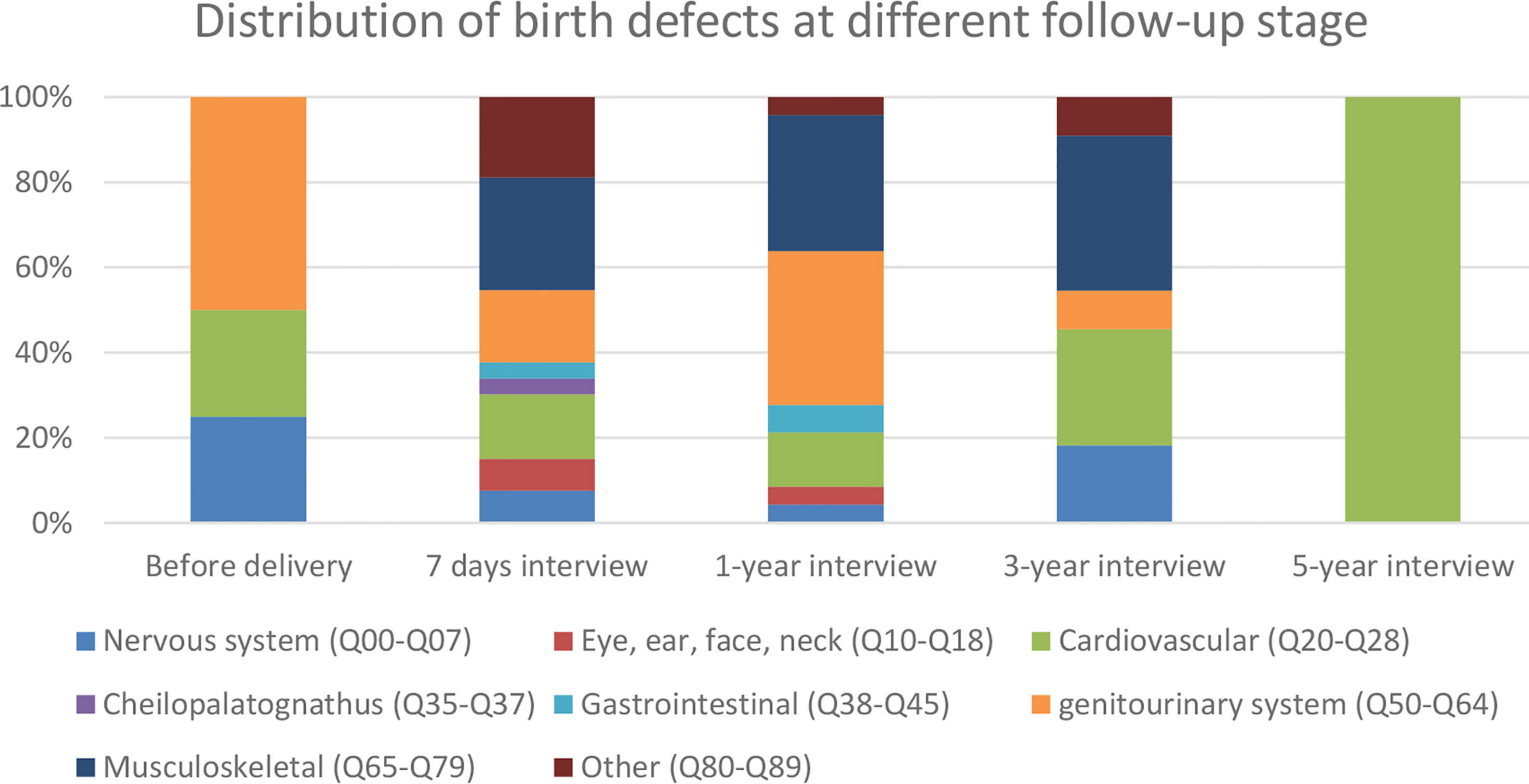
Distribution of birth defects at different follow-up stages.

50 babies (with 53 defects) were diagnosed at the 7-day follow-up. The main categories of congenital malformations included cardiovascular and circulatory malformations (8 cases); genitourinary malformations (9 cases), musculoskeletal malformations (14 cases), and other malformations (10 cases). Details of birth defects are listed in **Supplement 2** and **Figure 3**.

Forty percent of the cases (46 cases, 47 defects) were diagnosed at the 1-year follow-up. The categories of new congenital malformations at this stage included cardiovascular and circulation malformations (6 cases), genitourinary malformations (17 cases), and musculoskeletal malformations (15 cases). Only 11 cases with defects were diagnosed after 1 year. The categories included cardiovascular and circulation malformations and musculoskeletal malformations (**Supplement 2** and **Figure 3**).

## Discussion

The reported birth defect rates after ART vary in the published literature (3, 5, 6, 8, 9). Different definitions of birth defects (6, 17) and different follow-up durations (11–14) likely contribute substantially to the discrepancies. This makes direct comparison of their data difficult.

3.4% (4/117) of defects were diagnosed prenatally in our study; however, in a previous literature from South Australia, the corresponding rate was up to 18.7% (11). The low incidence at this stage of our study was due to two reasons. First is the definition of perinatal birth defects in China (15), which is defined as 28 weeks of gestation to 7 days after birth, whereas in South Australia, all live births and stillbirths of at least 20 weeks’ gestation or with a birth weight of at least 400 g are recorded as birth defect with the use of a standardized notification form (17). Second is well-developed prenatal screening systems; these made chromosomal abnormalities such as town’s syndrome be screened by non-invasive prenatal testing (18). Furthermore, through the standard prenatal ultrasonic examination, some structural defects could be discovered before 24 weeks (19), and most of the women underwent termination of pregnancy immediately after diagnosis in China. These strategies made only four fetuses diagnosed birth defect before delivery; two terminated after 28 weeks, the other two delivered and operated on after delivery.

Similar with previous studies (6, 14), most of the physically obvious defects defined as congenital malformation are usually diagnosed immediately after birth. The mainly species are those easily visible, such as cheilopalatognathus, polydactylism, and cutaneous hemangioma. However, many birth defects, especially functional defects, could not be diagnosed at this stage; it reminded the necessity to prolong follow-up duration.

In our study, 40% of all birth defects (47/117) were not recognized until after the 7-day follow-up. Examples of these defects include abdominal wall defects and cryptorchidism. As more than 40% birth defects were identified between birth to 1 year, we believe that the data presented suggest that 1-year follow-up should be the minimum requirement for future studies of birth defects after ART. If registries only collected data to 7 days after delivery, a range of cardiovascular, genitourinary system and musculoskeletal system defects would potentially be missed.

After the 1-year assessment, only 11 birth defects were noted, accounting for 11% of the overall birth defects. Among them, half were cardiovascular defects; at 3 years after delivery, the cumulative prevalence of birth defects was 5.5% and 98.2% of all the defects eventually detected were documented.

We found a paucity of articles that reported on their LTFU rate. In our study, the LTFU at 7 days after delivery was 0.1% and the LTFU was 11%, and 19% by 1 and 3 years after delivery, respectively. By 5 years after delivery, the LTFU rate increases to 35.5% which is almost three times compared to 3 years after delivery. Therefore, in order to better understand birth defects after ART treatment, we believe that 1-year follow-up after birth is the minimum requirement in ART treatment and 3-year follow-up is the optimal duration that allows for >95% ascertainment of all birth defects eventually diagnosed.

There are some limitations of our study. Although we collected data from three hospitals over 5 years, a total of 1,985 babies may be not enough to detect some birth defects. Secondly, the fact that we obtained the information of birth defects from parents not from a professional pediatrician might affect the accuracy of the description of the specific diagnosis. Thirdly, accompanied by those loss-to-follow-up cases, some birth defects might be missed in data collection. The actual birth defect rate may be a little higher. In conclusion, we suggest that in ART, 1-year follow-up pulse should be the minimum requirement and 3-year follow-up the optimal follow-up interval for ascertaining the risk of birth defects.

## Data Availability Statement

The original contributions presented in the study are included in the article/**Supplementary Material**. Further inquiries can be directed to the corresponding author.

## Ethics Statement

The studies involving human participants were reviewed and approved by the Reproductive Medical Ethics Committee of Guangzhou Women and Children’s Medical Center. The patients/participants provided their written informed consent to participate in this study. Written informed consent was obtained from the individual(s) for the publication of any potentially identifiable images or data included in this article.

## Author Contributions

LS was responsible for the conception and design of the study as well as analysis and interpretation of data; prepared, drafted, and revised the article critically for important intellectual content; and approved the final draft for publication. C-LL, PL, and G-FC contributed to collection of the data, drafting and revision of the entire article, and analysis and interpretation of data. AM contributed to revision of the article for important intellectual content and editing of the English grammar. Z-HC, JL, and XZ contributed to collection of the data, as well as drafting and revision of the article for important intellectual content. All authors contributed to the article and approved the submitted version.

## Conflict of Interest

The authors declare that the research was conducted in the absence of any commercial or financial relationships that could be construed as a potential conflict of interest.

## Publisher’s Note

All claims expressed in this article are solely those of the authors and do not necessarily represent those of their affiliated organizations, or those of the publisher, the editors and the reviewers. Any product that may be evaluated in this article, or claim that may be made by its manufacturer, is not guaranteed or endorsed by the publisher.
