# Supplementary material for: Optimal Follow-Up Duration for Assessment of Birth Defects After In Vitro Fertilization–Embryo Transfer: A Multicenter 5-Year Cohort Study in China
Source: Front Endocrinol (Lausanne). 2022 Mar 18;13:817397. doi: 10.3389/fendo.2022.817397 (PMC8971599; doi:10.3389/fendo.2022.817397)
Supplement: Supplementary file 1 [file DataSheet_1.pdf]

Supplement 1:

Contents of follow-up phone call by length of time since termination of delivery

Duration of follow-up	Questionnaire of follow-up
Before delivery	1) Progressive pregnancy: ☐yes , ☐no ; If “No” was answered, please complete the following question.<br>Reasons of termination:
7 days after Delivery	1) Maternal complications: ☐yes , ☐no ; If “Yes” was answered, please complete following question.<br>☐abnormal of amniotic fluid, ☐abnormal of placenta, ☐gestational hypertension,<br>☐gestational diabetes, ☐preterm delivery, ☐others<br>2) Neonatal complications: ☐yes , ☐no ; If “Yes” was answered, please complete following question.<br>☐neonatal respiratory distress syndrome (NRDS), ☐asphyxia, ☐infection、☐Intracranial hemorrhage, ☐ pathological jaundice, ☐others<br>3) Neonatal birth defects: ☐yes , ☐no ; If “Yes” was answered, please complete following question.<br>☐congenital heart disease, ☐genitourinary malformations, ☐nervous system malformations,<br>☐gastrointestinal malformations, ☐others
1 /3 /5-years after Delivery	1) birth defects: ☐yes , ☐no ; If “Yes” was answered, please complete following question.<br>☐congenital heart disease, ☐genitourinary malformations, ☐nervous system malformations,<br>☐gastrointestinal malformations, ☐others<br>2) when the malformation was diagnosed:<br>3) treatment methods:

number
