# Supplementary material for: Optimal Follow-Up Duration for Assessment of Birth Defects After In Vitro Fertilization–Embryo Transfer: A Multicenter 5-Year Cohort Study in China
Source: Front Endocrinol (Lausanne). 2022 Mar 18;13:817397. doi: 10.3389/fendo.2022.817397 (PMC8971599; doi:10.3389/fendo.2022.817397)
Supplement: Supplementary file 2 [file DataSheet_2.pdf]

<u>Supplement 2:</u>

Detail categories of birth defects at different duration of interview

Diagnostic category	Before delivery	7 days interview	1-year interview	3-year interview	5-year interview
	Fetuses: N=4<br>Defects: N=4	Babies: N=50<br>Defects: N=53	Babies: N=46<br>Defects: N=47	Babies: N=9<br>Defects: N=11	Babies: N=2<br>Defects: N=2
Nervous system (Q00-Q07)	1	4	2	2	
Eye, ear, face, neck (Q10-Q18)		4	2		
Cardiovascular (Q20-Q28)	1	8	6	4	2
Cheilopalatognathus (Q35-Q37)		2			
Gastrointestinal (Q38-Q45)		2	3		
genitourinary system (Q50-Q64)	2	9	17	1	
Musculoskeletal (Q65-Q79)		14	15	3	
Other (Q80-Q89)		10	2	1	
Chromosomal (Q90-Q99)					
